# Risk factors for nosocomial rectal colonization with carbapenem-resistant *Acinetobacter baumannii* in hospital: a matched case–control study

**DOI:** 10.1186/s13756-021-00919-6

**Published:** 2021-04-08

**Authors:** Marianna Meschiari, Shaniko Kaleci, Gabriella Orlando, Silvia Selmi, Antonella Santoro, Erica Bacca, Marianna Menozzi, Erica Franceschini, Cinzia Puzzolante, Andrea Bedini, Mario Sarti, Claudia Venturelli, Elena Vecchi, Cristina Mussini

**Affiliations:** 1 Department of infectious Diseases, Azienda Ospedaliero-Universitaria, University of Modena and Reggio Emilia, Via del Pozzo 71, 41122 Modena, Italy; 2 Clinical and Experimental Medicine, University of Modena and Reggio Emilia, Modena, Italy; 3 Clinical Microbiology Laboratory, University of Modena and Reggio Emilia, Modena, Italy; 4 Hospital Hygiene and Infection Control, Azienda Ospedaliero-Universitaria, Modena, Italy

**Keywords:** Carbapenem-resistant *Acinetobacter baumannii*, Rectal colonization, Surveillance, Risk factors, Endemic hospital

## Abstract

**Background:**

During the last decade carbapenem-resistant *Acinetobacter baumannii* (CRAB) became hyper-endemic in hospitals due to difficult to control spreading. Our aim is to identify risk factors for nosocomial rectal CRAB colonization in an endemic hospital.

**Methods:**

A retrospective matched case–control study (ratio 1:2) with a prospective inclusion of cases and concurrent selection of controls was conducted from January 2017 to December 2018 in a tertiary-care hospital. Universal active surveillance for CRAB was implemented. Univariate and multivariate logistic regression was carried out using a stepwise selection method to compare prognostic factors between cases and controls. A sub-analysis was carried out according to the type of department.

**Results:**

Forty-five cases with nosocomial rectal CRAB colonization and 90 controls were included. One hundred and two (75%) patients were hospitalized in medical departments. At multivariable analysis significant risk factors associated with CRAB colonization were: use of permanent devices (OR 10.15, 95% CI 2.27–45.39; *P* = 0.002), mechanical ventilation (OR 40.01, 95% CI 4.05–395.1; *P* = 0.002), urinary catheters (OR 4.9, 95% CI 1.52–16.19; *P* = 0.008), McCabe score (OR 5.45, 95% CI 1.87–15.89; *P* = 0.002), length of stay (OR 1.03, 95% CI 1.01–1.05; *P* = 0.002), carbapenem use (OR 5.39, 95% CI 1.14–25.44; *P* = 0.033). The sub-analysis showed that patients admitted to different departments had different risk factors. In geriatric department a fatal disease and a longer hospital stay represented significant risk factors both in univariate and multivariate analysis, while in internal medicine department the use of permanent devices, current antibiotic therapy and antibiotic polytherapy represented significant risk factors for CRAB at the univariate analysis, also confirmed in multivariate analysis.

**Conclusions:**

Our data suggest that active surveillance for rectal CRAB colonization should be addressed to patients with an unfavourable prognosis, longer hospitalizations and carriers of multiple devices. To counter CRAB spreading in endemic settings, clinicians must limit the use of carbapenems, and reinforce interventions aimed at proper use of devices.

## Background

Carbapenem-resistant *Acinetobacter baumannii* (CRAB) is emerging worldwide as a major cause of healthcare-associated infections (HAIs), especially in intensive care units (ICUs) [1–4]. Worryingly, during the last decade, the Italian antimicrobial resistance rates were among the highest in Europe and CRAB became hyper-endemic [5]. Its growing importance in the hospital setting is due both to the ability of these bacteria to accumulate mechanisms of resistance to antibiotics, and the ability to survive in unfavourable conditions for long periods of time [6]. CRAB infections frequently occur in patients with severe underlying diseases, the critically ill, the elderly, immunocompromised, severely debilitated or with life-threatening conditions and also in patients transferred from long-term health care facilities (LTHCFs) [7, 8]. Risk factors mainly associated with CRAB infection and colonization are invasive procedures, indwelling devices and previous antibiotic treatment [9]. Moreover, cross-transmission of CRAB among hospitalized patients is promoted by poor adherence to hand hygiene practices and by repeated contact with the contaminated environment [4, 10–13]. Therefore, the rapid identification of CRAB asymptomatic carrier could allow an earlier introduction of infection contact precautions to prevent transmission to other patients and to the hospital environment [14]. An early recognition of CRAB carrier can also assist to identifying patients at risk of subsequent CRAB infection. Indeed, Latibeaudiere et al. demonstrated that previous CRAB colonization increased by 8 times the risk to develop a CRAB infection [4, 15]. Several studies have been carried out worldwide with the aim of identifying risk factors for colonization and infection with *A. baumannii*, in particular focusing on CRAB [16–19]. Unfortunately, most of these studies were retrospective and conducted during CRAB outbreaks. Furthermore, many studies were limited to ICUs where colonization and infections with *Acinetobacter spp.* are more frequent [1, 16, 20, 21], while only a few studies investigated non-ICUs settings [3, 19]. Only two studies involved LTHCFs [17, 22]. The most common outcome was risk factors for CRAB infection and subsequent mortality. These heterogeneous studies conducted in different epidemiological situations had methodological limitations mainly due to different selection criteria between cases and controls, and did not allow conclusive findings [1, 20, 23, 24]. This study aims to identify the main risk factors associated with rectal CRAB nosocomial colonization in a CRAB-endemic acute care facility.

## Methods

### Setting and definitions

The study was conducted from January 2017 to December 2018 at the Modena University Hospital, a tertiary-care hospital with 1200 beds. The region is endemic for CRAB, thus, according to hospital infection control policy, a universal active surveillance was implemented during the study period with a rectal swab performed at hospital admission and repeated weekly in the whole hospital. Here in details our surveillance protocol: since the beginning of 2014 due to the increasing rate of Carbapenem-resistant Gram-negative organisms (CRGNOs), we decided to abolish target screening, based on specific risk factors contained in the regional chart, in favor of the introduction of mandatory universal screening on admission and repeated weekly according to the result obtained by wo serial point prevalence survey. We divided the whole hospital in high-risk wards (with mandatory universal screening on admission and weekly screening for contacts) and low-risk ones (with non-systematic screening on admission, but with weekly contacts screening only in case of positive carrier); a carrier was considered positive until discharge, and for almost a year after first CRGNOs isolate.

Contact precautions for all CRGNOs including CRAB infected patients and asymptomatic carriers included: single room, cohorting or spatial isolation; alert code out of the rooms and on the bed, staff wear gown and gloves upon entry to a room; single-use or patient-dedicated non-critical care equipment.

A matched case–control study with a prospective inclusion of cases and concurrent selection of controls (ratio 1:2) was designed. A patient was defined as a case meeting all of the following criteria: nosocomial isolation of a CRAB strain from rectal swab screening (isolated ≥ 72 h from admission time), a negative rectal swab at hospital admission and no isolation of CRAB from any biological sample in the previous 6 months. Two controls were individually matched to each case by age, date of screening and department at the time of screening. Controls were selected among patients with a negative rectal swab for CRAB on admission and on the same day as the matched case. Similar to cases, controls had a hospital stay at time of screening longer than 4 days and no isolation of CRAB from any biological sample in the previous 6 months.

Several risk factors, based on previous literature studies were investigated and included both patient-related risk factors, such as age or the presence of comorbidities and extrinsic risk factors related to patient hospitalization. The following data were collected: age, sex, length of hospital stay (LOS) at time of positive screening, previous ICU stay, provenance of patients at admission, overall mortality at 90 days, previous hospitalizations within 6 months, comorbidities, assessed by the Charlson Comorbidity Index (CCI) and specific covariates composing the index [25], McCabe prognostic classification score [26], disabilities defined as independence in activities of daily living, permanent devices (including indwelling urinary catheters), exposure to invasive devices and medical procedures during hospitalization, immunosuppressive therapies (chemotherapy or minimum dose of 0.3 mg/kg/day of prednisone equivalent for > 3 weeks) and antibiotic administration prior CRAB colonization distinguishing between before and during the hospitalization (≤ 30 days before the admission). Antibiotic polytherapy was defined as two or more classes of antibiotics simultaneously prescribed. The size of the study sample was calculated to detect associations with an odds ratio (OR) = 3.5, considering an error of 5%, power of 80%, and proportion of exposed controls of 25%, and allowing for the matched study design. A specific study form has been used to collect data from hospital medical charts. The microbiology laboratory provided the list of potential matched controls for each identified case, ordered by the laboratory request code of the screening test. The list included all patients meeting the criteria for controls described above. All eligible cases and 2 matched controls were selected consecutively. This study was approved by the Modena University Hospital Institutional Ethics Committee with the following approval number: AOU 0025972/19 on the 25/09/2019. No written informed consent was provided to patients as all data were analysed anonymously after a de-identification process.

### Microbiological methods

All isolates were identified by MALDI-TOF MS using the VITEK MS (bioMérieux, Marcy l´Etoile, France) following the manufacturer’s instructions. Antimicrobial susceptibility testing was performed by microdilution method using the antimicrobial susceptibility testing ITGNEGF panel (MICRONAUT, Merlin, Germany).

### Statistical analysis

Descriptive statistics were performed for baseline demographic clinical characteristics of the entire group, as well as the groups of patients with and without colonization with CRAB. Continuous variables were presented as the number of patients (N), mean, standard deviation (SD). Unpaired Student's t test was used to compare groups. Categorical variables were presented as frequency (N, percentage [%]) and compared using Pearson's chi-squared test (Fisher’s exact test was used for those variables with less than 5 events). A multivariate logistic regression model was carried out using a stepwise selection method to identify the prognostic factors between cases and controls. In the first step, the intercept-only model was fitted and individual score statistics for the potential variables were evaluated. A significance level of *P* < 0.05 was used to allow a variable into the model. In stepwise selection, an attempt was made to remove any insignificant variables from the model before adding a significant variable to the model. Hosmer and Lemeshow tests were used to evaluate “goodness of fit” in the selection model. Data from the univariate and multivariate logistic regression analyses were expressed as odds ratio (OR) and 95% confidence interval (CI). A *P* < 0.05 was considered statistically significant. Statistical analysis was performed using STATA® software version 14 (StataCorp. 2015. Stata Statistical Software: Release 14. College Station, TX: StataCorp LP.).

## Results

In this study, 45 (33.4%) patients colonized with CRAB were considered as cases; for each patient two controls were selected up to a total of 90 (66.6%) controls. One hundred and two (75.5%) patients were hospitalized in medical departments, including geriatric and internal medicine, 21 (15.5%) patients in ICUs and 12 (9%) in surgical departments. The baseline characteristics of the two matching groups and the comparisons are described in Table 1.

**Table 1:** Demographic and clinical characteristics of patients colonized with CRAB and matched controls

Variables	Controls		Cases		Total		*P* value
	N = 90 (66.6%)		N = 45 (33.4%)		N = 135		
	N	%	N	%	N	%	
Age (years), Mean ± SD (range)	76 ± 15 (18–94)		75 ± 16 (18–95)		75.7 ± 16 (18–95)		0.769
Male, sex	59	65.6	26	57.8	85	63	0.378
LOS, mean ± SD (range)	26.8 ± 23.3 (3–127)		48.7 ± 34.0 (7–159)		34.1 ± 29.1 (3–159)		< 0.001
ICU stay	18	20	20	44.4	38	28.1	0.003
^a^Deaths	1	2.9	5	29.4	6	11.8	0.006
*Provenance of patient at admission*							
Home	83	92.2	36	80.0	119	88.2	0.003
LTHCF	1	1.1	7	15.6	8	5.9	
Other hospital	6	6.7	2	4.4	8	5.9	
^b^Recent hospitalization	6	6.7	8	17.8	14	10.4	0.046
*Charlson Comorbidity Index,* Mean ± SD (range)	5.9 ± 2.5 (0–12)	6.9 ± 2.7 (0–12)	6.3 ± 2.6(0–12)	0.050			
*McCabe score*							
Nonfatal disease	54	60.0	20	44.4	74	54.8	0.019
Fatal disease (within 5 years)	36	40.0	22	48.9	58	43.0	
Rapidly fatal disease (within 6 months)	0	0.0	3	6.7	3	2.2	
Major surgery ≤ 30 days before hospitalization	3	3.3	2	4.4	5	3.7	0.747
Major surgery during hospitalization	20	22.2	15	33.3	35	25.9	0.165
*Diagnosis at hospital admission*							
Infection	54	60.0	32	71.1	86	63.7	0.115
Polytrauma	17	18.9	2	4.4	19	14.1	
Cardiovascular disease	15	16.7	10	22.2	25	18.5	
Cancer	4	4.4	1	2.2	5	3.7	
*Presence of intrinsic risk factors and comorbidities*							
Chronic heart failure	48	53.3	28	62.2	76	56.3	0.326
Hypertension	57	63.3	29	64.4	86	63.7	0.899
Peripheral vascular disease	22	24.4	20	44.4	42	31.1	0.018
Stroke or TIA	27	30.0	18	40.0	45	33.3	0.245
Dementia	22	24.4	18	40.0	40	29.6	0.062
COPD	16	17.8	10	22.2	26	19.3	0.537
Chronic hepatitis	8	8.9	3	6.7	11	8.1	0.656
Gastrointestinal disease	36	40.0	12	26.7	48	35.6	0.127
Solid neoplasia & haematological neoplasia	28	31.1	15	33.3	43	31.8	0.693
CKD	33	36.7	13	28.9	46	34.1	0.369
Diabetes mellitus	22	24.4	15	33.3	37	27.4	0.275
*Disability*		0.0		0.0		0	
No disability	68	75.6	22	48.9	90	66.7	0.003
Partial	15	16.7	11	24.4	26	19.3	
Bedridden	7	7.8	12	26.7	19	14.1	
*Permanent devices*	6	6.7	11	24.4	17	12.6	0.003
*Presence of extrinsic risk factors*							
Central vascular catheterization	7	7.8	21	46.7	28	20.7	< 0.001
PICC or midline	6	6.7	11	24.4	17	12.6	0.003
Urinary catheter	38	42.2	37	82.2	75	55.6	< 0.001
Naso-gastric tube	8	8.9	13	28.9	21	15.6	0.003
PEG	1	1.1	5	11.1	6	4.4	0.008
Tracheostomy	3	3.3	12	26.7	15	11.1	< 0.001
Mechanical ventilation	7	7.8	10	22.2	17	12.6	0.017
Dialysis	3	3.3	3	6.7	6	4.4	0.376
Blood transfusion	24	26.7	11	24.4	35	25.9	0.781
Antibiotics ≤ 30 days before hospitalization	9	10.0	9	20.0	18	13.3	0.113
Chemotherapy	3	3.3	6	13.3	9	6.7	0.028
Corticosteroid therapy	15	16.7	16	35.6	31	23	0.014
^c^Antibiotics during hospitalization	65	72.2	43	95.6	108	80	0.001
3GC	26	28.9	20	44.4	46	34.1	0.087
Carbapenems	5	5.6	13	28.9	18	13.3	< 0.001
Penicillins	47	52.2	31	68.9	78	57.8	0.086
Fluoroquinolones	13	14.4	12	26.7	25	18.5	0.097
Glycopeptides	11	12.2	15	33.3	26	19.3	0.004
^d^Polytherapy	22	24.4	26	57.8	48	35.6	< 0.001
Number of antibiotics used in the hospitalization, mean ± SD (range)	1.53 ± 1.44 (0–5)		3.64 ± 2.69 (0–13)		2.24 ± 2.19 (0–13)		< 0.001

SD, standard deviation; LOS, Length of hospital stay; ICU, Intensive care unit; LTHCF, Long-term health care facility; TIA, transient ischemic attack; COPD, Chronic obstructive pulmonary disease; CKD, chronic kidney disease; PICC, Peripherally inserted central catheter; PEG, Percutaneous endoscopic gastrostomy; NIV, Noninvasive ventilation

^a^The number of deaths was defined as all-cause mortality within 90 days of hospital admission

^b^Previous hospitalization within 6 months

^c^Antibiotic exposures spanned from hospital admission to development of CRAB colonization

^d^Therapy with 2 or more concurrent antibiotic classes before developing CRAB colonization

Several parameters proved to be statistically significant risk factors in the univariate analysis (Table 2). An ICU admission during hospitalization increased the risk of acquiring a rectal colonization with CRAB in hospital by 3.2 times, while patients transferred from a LTHCF had a 16 time higher risk of nosocomial rectal CRAB colonization compared to patients living at home. Considering comorbidities, peripheral cardiovascular disease and dementia increased the risk of being colonized with CRAB. Other variables significantly associated to risk of rectal colonization with CRAB were the use of permanent devices and the use of invasive devices in a hospital environment. Among these were the use of vascular catheters, such as central venous catheterization (CVC) or the use of Peripherally Inserted Central Catheter (PICC) or Midline, urinary catheter (UC), nasogastric tube (NG) and mechanical ventilation (MV). Other factors that were significantly associated were tracheostomy and percutaneous endoscopic gastrostomy (PEG), which respectively determined an elevated risk for rectal CRAB colonization of 10.5 and 11 times.

**Table 2:** Univariate and multivariate analysis of risk factors and outcomes related to CRAB colonization

Variables	Univariate analysis			Multivariate analysis		
	OR	95% CI	*P* value	OR	95% CI	*P* value
Age (years)	0.99	0.97–1.01	0.767			
Sex, male	0.71	0.34–1.49	0.378			
LOS	1.02	1.01–1.04	< 0.001	1.03	1.01–1.05	0.002
ICU stay	3.2	1.46–6.99	0.004			
^a^Deaths	5.5	1.77–17.01	0.003			
*Provenance of patient at admission*						
Home	Ref.					
LTHCF	16.14	1.91–136	0.011			
Other hospital	0.76	0.14–3.99	0.754			
Recent hospitalization	3.02	0.98–9.34	0.054			
*Charlson Comorbidity Index*	1.15	0.99–1.33	0.053			
*McCabe score, nonfatal vs. fatal disease and rapidly fatal disease*	2.12	1.08–4.14	0.027	5.45	1.87–15.89	0.002
Major surgery ≤ 30 days before hospitalization	1.34	0.21–8.37	0.748			
Major surgery during hospitalization	1.75	0.79–3.87	0.167			
*Diagnosis at hospital admission*						
Infection	ref					
Polytrauma	0.19	0.04–0.91	0.038			
Cardiovascular disease	1.12	0.45–2.79	0.800			
Cancer	0.42	0.0.4–3.94	0.449			
*Presence of intrinsic risk factors and comorbidity*						
Chronic heart failure	1.44	0.69–2.99	0.327			
Hypertension	1.04	0.49–2.21	0.899			
Peripheral vascular disease	2.47	1.15–5.28	0.019			
Stroke or TIA	1.55	0.73–3.28	0.247			
Dementia	2.06	0.95–4.43	0.064			
COPD	1.32	0.54–3.20	0.538			
Chronic hepatitis, cirrhosis	0.73	0.18–2.90	0.657			
Gastrointestinal disease	0.54	0.24–1.19	0.130			
Solid neoplasia	1.10	0.51–2.37	0.794			
CKD	0.70	0.32–1.52	0.370			
Diabetes mellitus	1.54	0.70–3.38	0.277			
*Disability*						
No disability	ref					
Partial	2.26	0.90–5.65	0.79			
Bedridden	5.29	1.85–15.12	0.002			
*Permanent devices*	4.52	1.55–13.22	0.006	10.15	2.27–45.39	0.002
*Presence of extrinsic risk factors*						
Central vascular catheterization	10.37	3.93–27.32	< 0.001			
PICC or midline	4.52	1.55–13.22	0.006			
Urinary catheter	7.23	2.91–17.96	< 0.001	4.96	1.52–16.19	0.008
Naso-gastric tube	4.16	1.57–10.99	0.004			
PEG	11.12	1.25–98–33	0.030			
Tracheostomy	10.54	2.79–39.75	0.001			
Mechanical ventilation	3.38	1.19–9.61	0.022	40.01	4.05–395.1	0.002
Dialysis	2.07	0.40–10.70	0.385			
Blood transfusion	0.88	0.38–2.03	0.781			
Antibiotics ≤ 30 days before hospitalization	2.22	0.81–6.06	0.119			
Chemotherapy	4.46	1.06–18.76	0.041			
Corticosteroid therapy	2.75	1.20–6.29	0.016			
Antibiotics during hospitalization	8.26	1.86–36–72	0.005			
3GC	1.90	0.90–4.01	0.089			
Carbapenems	6.66	2.19–20.20	0.001	5.39	1.14–25.44	0.033
Penicillins	1.93	0.90–4.11	0.088			
Fluoroquinolones	2.09	0.86–5.08	0.101			
Glycopeptides	3.5	1.44–8.48	0.006			
Polytherapy	4.65	2.13–10.14	< 0.001			
Number of antibiotics used in the hospitalization, mean ± SD (range)	1.73	1.36–2.19	< 0.001			

CI, confidence interval; OR, odds ratio

^a^Model for multivariable logistic regression included surveillance status, mechanical ventilation, sex, and exposure to any antibiotic (fit criteria quasi-likelihood criterion [QIC] = 336)

Concerning therapy, the use of antibiotics and antibiotic polytherapy during hospitalization was found to be very strong risk factors in the univariate analysis. With regard to the different antibiotic classes, carbapenems and glycopeptides statistically increased the rate of rectal colonization with CRAB, but not the use of 3rd generation Cephalosporins (3GC). Moreover, corticosteroid therapy was found to be a significant risk factor for CRAB. In the multivariate logistic regression analysis shown in Table 2, a significant independent risk factor for rectal CRAB colonization was the use of permanent devices. The use of UC and MV increased the risk by 5 and 40 times respectively. McCabe score elevated the risk for CRAB rectal colonization by 5.45 times. Another significant risk factor was LOS. Among antibiotic exposure, only carbapenems were significantly associated with rectal CRAB. Finally, there was no association with mortality.

### Subgroup analysis by department

A subgroup analysis to identify specific risk factors for rectal CRAB colonization in the different departments was performed (Table 3). We categorized the data according to the department in which the patient was hospitalized at the time of screening positive for CRAB.

**Table 3:** Variables associated with CRAB colonization between different departments: geriatrics, internal medicine and ICU

Variables	Geriatrics n = 51			Internal medicine = 51			ICU n = 21		
	Univariate analysis			Univariate analysis			Univariate analysis		
	OR	95% CI	*P* value	OR	95% CI	*P* value	OR	95% CI	*P* value
Age (years)	0.99	0.91–1.08	0.896	0.99	0.95–1.04	0.861	0.99	0.95–1.03	0.843
Sex, male	0.77	0.23–2.57	0.682	0.49	0.15–1.59	0.237	1	0.07–13.36	1.000
LOS	1.01*	0.99–1.03	0.082	1.02*	1.00–1.05	0.012	1.03	0.99–1.07	0.134
ICU stay	7.07	0.67–73.99	0.103	6.52	1.70–25.03	0.006	2.4	0.21–26.82	0.477
^a^Deaths	13.75	1.45–129.98	0.022	1	0.08–11.87	1.000	8	0.96–66.44	0.054
*Provenance of patient at admission*									
Home	ref.			ref.			ref.		
LTHCF	–			4.46	0.37–53.70	0.239	–		
Other hospital	–			1.11	0.18–6.87	0.906	–		
Recent hospitalization	1	0.08–11.87	1.000	3.17	0.62–16.24	0.165	9.75	0.78–121.83	0.077
*Charlson index*	1.15	0.84–1.57	0.358	1.21	0.95–1.55	0.113	1.18	0.83–1.67	0.335
*McCabe Score, Nonfatal vs. fatal disease and rapidly fatal disease*	2.69*	0.80–8.93	0.107	0.76	0.21–2.68	0.675	8.38	1.16–60.45	0.035
Major surgery during hospitalization	0.69	0.15–3.05	0.631	4.30	0.88–20.87	0.070	4.49	0.54–37.37	0.164
*Disability*									
No disability	ref.			ref.			ref.		
Partial	2.18	0.51–9.22		1.93	0.38–9.72	0.424	11	0.81–147.86	0.070
Bedridden	4.19	0.94–18–70		1	–		1.83	0.12–27.79	
*Permanent devices*				5.63*	1.19–26.48	0.028	1	–	
*Presence of extrinsic risk factors*									
Central vascular catheterization	6.66	1.13–39.09	0.036	18	3.23–100.21	0.001	1	–	
PICC or midline	1	–		1.60	0.31–8.16	0.567	4.49	0.54–37.37	0.164
Urinary catheter	10.71*	2.10–54.45	0.004	13.75	2.68–70.49	0.002	2.4	0.21–26.82	0.477
Naso-gastric tube	10.15	1.03–99.60	0.047	4.92	0.80–30.25	0.085	6.25	0.83–46.56	0.074
Mechanical ventilation	1	0.08–11.87	1.000	13.75	1.45–129.98	0.022	3.33	0.50–22.14	0.213
Antibiotics ≤ 30 days before hospitalization	3.02	0.68–13.22	0.142	1.37	0.20–9.14	0.740	2.4	0.26–22.10	0.440
Chemotherapy	2.13	0.27–16.63	0.470	4.39	0.36–52.37	0.241	1	–	
Corticosteroid therapy	3.26	0.88–12.08	0.076	1.60	0.31–8.16	0.567	6.25	0.83–46.56	0.074
*Antibiotics during hospitalization*	2.75	0.29–25.70	0.373	12.63*	1.50–106.36	0.020	1	–	
3GC	1.19	0.37–3.85	0.765	1	0.21–4.60	1.00	32.9	2.45–443.59	0.008
Carbapenems	3.32	0.49–22.14	0.215	8.72	153–49.75	0.015	14.66	1.16–185.23	0.038
Penicillins	0.77	0.23–2.57	0.682	4.64	1.24–17.24	0.022	1.2	0.08–16.23	0.891
Fluoroquinolones	1.71	0.39–7.49	0.468	3.17	0.62–16.24	0.165	1.2	0.18–7.77	0.848
Glycopeptides	4.06	1.04–15.72	0.043	2.13	0.27–16.63	0.470	7.5	0.92–61.04	0.060
Polytherapy	2.68	0.79–9.11	0.113	7.45	1.89–29.34	0.004	1	–	
Number of antibiotics used in the hospitalization, mean ± SD (range)	1.42	0.98–2.04	0.057	1.98	1.28–3.05	0.002	3.01	0.93–9.73	0.065

*Bold variables with confirmed *P* < 0.05 also in the multivariate analysis

#### Geriatric department

In 51 patients from the geriatric divisions all-cause mortality among cases was significantly higher than controls (29.4% vs. 2.9%, *P* < 0.05), as well as the average quantity of antibiotics used during the hospital stay (data not shown). Devices as UCs and CVCs represented significant risk factors at univariate analysis and the use of UC was also confirmed at multivariate analysis. A fatal disease and a longer LOS represented significant risk factors both in univariate and multivariate analysis. Glycopeptides was the only statistically significant antibiotic class associated with CRAB colonization.

#### Internal medicine department

Concerning 51 patients from the internal medicine department, among CRAB cases there were a significantly higher percentage of patients with partial disabilities or bedridden status compared to controls (data not shown). There were also significant differences between cases and controls regarding the use of devices, of MV, and antibiotics during hospitalization, antibiotic polytherapy (data not shown), and the average number of antibiotics administered during hospitalization (cases 3.11 ± 2.29 and controls 1.03 ± 1.24, *P* < 0.05). The univariate analysis showed that several variables had a statistically significant OR (Table 3). Prolonged hospitalization, previous admission to the ICU, MV, the use of permanent devices, and catheters during hospitalization represented important risk factors for rectal CRAB colonization for this specific population. Among these, only the use of permanent devices was confirmed as a significant risk factor at the multivariate analysis. Moreover, current antibiotic therapy and antibiotic polytherapy represented a significant risk factor for CRAB at the univariate analysis, also confirmed in multivariate analysis. In particular, the exposure to carbapenems and piperacillin/tazobactam significantly increased the risk of CRAB colonization by 9 and 5 times, respectively, as well as the higher number of antibiotics use (Table 3). Concerning comorbidities, only peripheral vascular disease was a risk factor for rectal colonisation with CRAB in internal medicine (OR 4.06, 95% CI 1.05–15.73, *P* = 0.043).

##### ICU

With regard to 7 patients acquiring rectal CRAB colonization in ICU vs 14 controls, some statistically significant differences were found (Table 3). The univariate analysis showed that the McCabe Score represented a significant risk factor for nosocomial CRAB colonization, a fatal or rapidly fatal disease increased the risk by 8 times for acquiring CRAB in ICU. Concerning antibiotic exposure, the use of 3GC and carbapenems increased the risk of colonisation by 33 times and 15 times respectively. Multivariate analysis was not carried out due to limited simple size.

#### General surgery department

Concerning the population admitted to surgical departments, we could not identify any statistically significant risk factor for this population due to the limited sample (n = 12) also affecting the statistical analysis.

## Discussion

Antibiotic therapy, disabilities and medical devices play a crucial role in rectal CRAB colonization. Our findings support active screening strategies targeted to early identification of CRAB asymptomatic carriers in an endemic hospital setting, including non-ICU departments, a previously underestimated setting. The early identification of specific risk factors for colonization with CRAB could become the cornerstone of a cost-saving prevention strategy. In our population, diagnosis of a fatal condition and longer LOS had the greatest impact on CRAB nosocomial acquisition, especially in elderly patients. Cases had a twofold longer LOS compared to control patients, suggesting that longer hospitalization could facilitate CRAB acquisition. The risk of CRAB colonization was increased by several factors: carbapenem exposure increased the risk by fivefold; permanent devices and MV increased the risk by 10 and 40 times respectively. Finally, among comorbidities and intrinsic risk factors [3], only peripheral vascular disease and dementia were found to be associated to CRAB colonization in our hospital setting. Several studies have shown that CRAB colonization represents the main significant risk factor for the development of CRAB-related infections, with very difficult therapeutic management due to limited antimicrobial treatment options, present and future [3, 15]. In this scenario, active surveillance cultures with rectal screening represent one of the most important strategies of a multimodal approach in order to counter CRAB hospital spreading in highly endemic settings [4]. To our knowledge, few studies have investigated the risk factors for colonization and most of them are focused on ICUs or aimed at highlighting the variables involved in the development of infection rather than colonization [19, 21, 23]. These limitations could be explained by the fact that rectal screening on admission is not universally performed outside the ICU, so it is very difficult to distinguish between community-associated and nosocomial colonization. Until now the identified risk factors for CRAB nosocomial acquisition are: disease severity measured by scores (e.g. APACHE II, McCabe), antibiotic use, invasive procedures such as catheterisations, enteral feeding or MV, ICU stay, and LOS [3, 16, 19, 21, 27]. Therefore, multiple and permanent devices should always set up a wake-up call to screen patients admitted in non-ICU departments in endemic hospitals. Although polytrauma, blood transfusions and major surgery are significant known risk factors for CRAB acquisition [16] the limited number of surgical patients in our study did not allow us to explore this finding. In our study only antibiotic polytherapy and exposure to carbapenems were found to be significant risk factors for CRAB colonization, in the overall study population. Antibiotic therapy, in particular fluoroquinolones, 3GC and aminoglycosides, both before and during hospitalization, have previously been identified as risk factors for rectal CRAB colonization and infection [3, 15, 16, 19, 21, 27]. Moghnieh et al., identified a colonization risk score for XDR A.baumannii in ICU patients, and confirmed the role of carbapenem and piperacillin-tazobactam exposure [21], while Tacconelli et al. [27], showed that methicillin-resistant Staphylococcus aureus isolation and beta-lactam use were among the independent risk factors for CRAB colonization and infection. Our study has several strengths. First, it focused on CRAB colonization and not systemic infection. This is extremely important in order to better identify risk factors for colonization by eliminating possible confounders associated with CRAB infection. Second, our study is the first to conduct a risk factor analysis according to the type of department, with a majority of patients from outside the ICU. The sub-analysis by department showed that patients admitted to different departments have different risk factors and that, as far as our centre is concerned, most of the colonization occurred in geriatric and internal medicine settings. We found that antibiotic exposure had a different effect depending on the department. In ICU, antibiotic use was the main risk factor for nosocomial CRAB colonization, and in particular the use of carbapenems and 3GC significantly increased the risk by 33 times and 14.7 times, respectively. In internal medicine department, the use of antibiotic polytherapy, carbapenems and penicillins (especially piperacillin/tazobactam) were among the most important risk factors for rectal CRAB colonization. The use of piperacillin/tazobactam has been poorly investigated in previous studies probably because most focused on ICU-CRAB carriers, while the highest consumption of this antibiotic occurs in medical department [3, 26]. Conversely, in the geriatric setting the most important risk factors to become a CRAB carrier were the presence of invasive devices, both permanent and positioned during the hospital stay, rather than antibiotic exposure. A third strong point of our study is its design. The matching by department and date of screening allowed us to control for the variation of CRAB colonization pressure and the cross-transmission in the department, which are potential confounders of the main associations considered in this study. In fact, the likelihood of a patient to receive an antibiotic prescription in a specific setting and the timing may be influenced by the perceived risk of a multidrug resistant organism (MDRO) infection, as physicians might be more prone to prescribe a broad spectrum antibiotic when other patients in the department are colonized or infected by MDROs. Furthermore, concurrent selection of controls provided an accurate estimate of relative risk even in case–control studies assessing non-rare outcomes. We excluded community-colonized patients through an active surveillance on admission. This study has some limitations. It is a single-centre retrospective study carried out in a CRAB endemic area. For this reason, our model may not be applicable to different settings, and the results must be generalized with caution. Furthermore, the design of the study did not allow us to consider some important parameters such as risk factors related to infection control strategies. Since the controls for each CRAB case were selected through a matching by department and date of screening, it was not possible to analyse the role that the roommate had in the acquisition of CRAB colonization. Another limitation of our study is the selection bias due to the case–control approach. We have not analysed all the patients colonized in the time frame considered, but 45 randomly selected patients who tested positive for rectal screening after at least 4 days of hospitalization. In addition, we excluded respiratory and urinary CRAB colonization because the retrospective nature of the study did not allow a precise discrimination between colonization and infection in these different specimens. Unfortunately, no specific risk factors were identified for the surgical department, because the surgical sample was too small. Further investigations are needed in this setting.

## Conclusions

As CRAB colonization is one of the most important risk factors for CRAB infection, targeted infection prevention and control (IPC) practices are crucial to limit CRAB spreading in hospitals, not only in ICUs, but also general departments where this pathogen is widely found. Rectal screening could represent a cornerstone to limit CRAB dissemination in endemic settings. By identifying the main risk factors associated to in-hospital rectal CRAB-acquisition in different wards, this study provides new real-life elements to limit CRAB cross-transmission and to target CRAB-IPC interventions. In the era of MDROs clinicians should strictly adhere to and apply bundles for permanent devices and invasive procedures management in order to target their use. Finally, our study supports the fundamental principles of antimicrobial stewardship, i.e. limiting the use of inappropriate antibiotic therapy, avoiding polytherapy and reducing carbapenem use in CRAB endemic hospitals.

## Data Availability

The datasets used and/or analysed during the current study are available from the corresponding author on reasonable request.
